# New Kunitz-Type HCRG Polypeptides from the Sea Anemone *Heteractis crispa*

**DOI:** 10.3390/md13106038

**Published:** 2015-09-24

**Authors:** Irina Gladkikh, Margarita Monastyrnaya, Elena Zelepuga, Oksana Sintsova, Valentin Tabakmakher, Oksana Gnedenko, Alexis Ivanov, Kuo-Feng Hua, Emma Kozlovskaya

**Affiliations:** 1 G.B. Elyakov Pacific Institute of Bioorganic Chemistry, Far Eastern Branch, Russian Academy of Sciences, 159, Pr. 100 let Vladivostoku, Vladivostok 690022, Russia; E-Mails: zel@piboc.dvo.ru (E.Z.); sintsova0@mail.ru (O.S.); tabval@yandex.ru (V.T.); kozempa@mail.ru (E.K.); 2 V.N. Orekhovich Institute of Biomedical Chemistry, Russian Academy of Sciences, 10, Pogodinskaya Street, Moscow 119121, Russia; E-Mails: oksana_gnedenko@pochta.ru (O.G.); asi@icnet.ru (A.I.); 3 Department of Biotechnology and Animal Science, National Ilan University, No. 1, Section 1, Shen-Lung road, Ilan 260, Taiwan; E-Mail: kuofenghua@gmail.com

**Keywords:** sea anemone, Kunitz-type protease inhibitors, structure, function, SPR, anti-inflammatory activity

## Abstract

Sea anemones are a rich source of Kunitz-type polypeptides that possess not only protease inhibitor activity, but also Kv channels toxicity, analgesic, antihistamine, and anti-inflammatory activities. Two Kunitz-type inhibitors belonging to a new *Heteractis crispa* RG (HCRG) polypeptide subfamily have been isolated from the sea anemone *Heteractis crispa*. The amino acid sequences of HCRG1 and HCRG2 identified using the Edman degradation method share up to 95% of their identity with the representatives of the HCGS polypeptide multigene subfamily derived from *H. crispa* cDNA. Polypeptides are characterized by positively charged Arg at the *N*-terminus as well as P1 Lys residue at their canonical binding loop, identical to those of bovine pancreatic trypsin inhibitor (BPTI). These polypeptides are shown by our current evidence to be more potent inhibitors of trypsin than the known representatives of the HCGS subfamily with P1Thr. The kinetic and thermodynamic characteristics of the intermolecular interactions between inhibitors and serine proteases were determined by the surface plasmon resonance (SPR) method. Residues functionally important for polypeptide binding to trypsin were revealed using molecular modeling methods. Furthermore, HCRG1 and HCRG2 possess anti-inflammatory activity, reducing tumor necrosis factor-α (TNF-α) and interleukin 6 (IL-6) secretions, as well as proIL-1β expression in lipopolysaccharide (LPS)-activated macrophages. However, there was no effect on nitric oxide (NO) generation.

## 1. Introduction

According to phylogenetic studies, sea anemones (phylum Cnidaria, class Anthozoa) are one of the oldest group of venomous marine animals [1]. Sea anemone venom contains a variety of different proteinaceous toxic components, which are particularly important for prey capture and defending against predators. Although they remain one of the most poorly studied venomous animal lineages, several sea anemone biologically active polypeptides have been characterized for their structurally and functionally properties. Among the most widely and extensively studied toxins are the neurotoxins (blockers or activators of potential-sensitive Kv and Nav channels including proton-sensitive ASIC3, 3–5 kDa [2,3,4,5]), actinoporins (membrane active pore-forming toxins, 16–20 kDa [6,7,8,9]), and serine protease inhibitors of Kunitz/BPTI family (6–7 kDa [2,10,11]). Serine protease inhibitors are less diverse structurally, but are capable of carrying out a wide variety of functions.

Genes encoding Kunitz-type polypeptides have evolved from a common ancestor, which is responsible for the serine protease binding not undergoing any significant changes [12]. Kunitz-type polypeptides contain one of the most evolutionarily ancient and the most conserved among the protein structural motifs, the Kunitz fold [10,11], which was first found in the bovine pancreatic trypsin inhibitor (BPTI) [13]. The representatives of this group form a compact and stable alpha+beta fold stabilized well by three conservatively positioned disulfide bridges with the bonding patterns C^1^–C^6^, C^2^–C^4^, C^3^–C^5^ [10]. The majority of Kunitz-type polypeptides from sea anemones have a relatively conserved binding loop with a P1 residue Arg or Lys, which is known to be essential for inhibition of trypsin-like proteases belong to the S1 family [13,14]. Nevertheless, mutations among the amino acid residues found at the P1 position (K → R → T) and other point mutations throughout the amino acid sequences resulted in the polypeptides interacting with different biological targets [15,16,17,18].

Sea anemone Kunitz-type polypeptides are active against serine (trypsin, chymotrypsin, kallikreins, elastase, cathepsin G), cysteine (papain, bromelain), and aspartic (chymosin, pepsin) proteases [19,20,21,22,23,24,25,26,27,28], which are involved in many physiological processes of living organisms such as digestion, and inflammation. RmInI and RmInII toxins from *Heteractis crispa* exhibit a P1 Lys, which possesses trypsin and chymotrypsin inhibitory as well as antihistamine activities *in vivo* [24]. Also within *H. crispa* atypical polypeptides Jn-IV [23] and InhVJ [25,26] have a Thr residue at the P1 position, which has been shown to make them highly specific inhibitors of trypsin and α-chymotrypsin. Three polypeptides from *H. crispa*, APHC1–APHC3, with P1Thr have been recently revealed not only to weakly block serine proteases but also to modulate the activity of the TRPV1 receptor *in vitro* and develop analgesic activity *in vivo* [29,30]. Polypeptides belonging to type 2 toxins, AsKC1–AsKC3, or kalicludines 1–3 [16], SHTX III [17], and APEKTx1 [18] possess both trypsin inhibiting and Kv1 channel modulating activities. Thus, the phenomenon of polyfunctionality is a characteristic feature of the sea anemone Kunitz-type polypeptides.

Recent evidence suggests that the Kunitz-type polypeptides for *H. crispa* are encoded by a multigene superfamily composed of distinct GS-, GG-, GN-, and RG-gene subfamilies which are produced in the sea anemone venom via a combinatorial library [15]. In total, 33 mature polypeptides of the HCGS subfamily have been discovered and categorized into three groups according to phylogenetic data and the nature of the P1 residues (Arg, Lys, or Thr) [15].

Protease inhibitors with the Kunitz domain(s) possess such important properties as participation in anti-inflammatory processes including inhibition of inflammatory proteases, modulation of cytokine expression and signal transduction, tissue remodeling, and many others [31]. Endogenous inhibitor, such as BPTI in the form of aprotinin or Trasylol [32], is one of the most studied polypeptide of the Kunitz type. Despite the obvious anti-inflammatory activity, its operation is limited by some side effects as allergy and anaphylaxis. Sea anemone Kunitz-type polypeptides possess both anti-inflammatory and antihistamine activity [24,33], so are possibly able to overcome these negative effects. The investigation of structures and functions of Kunitz-type polypeptides, in particular HCRG subfamily representatives, both native and derived from the structure of coding genes, is not only an important practical task but also a fundamental one. As new data on the structure and function of the representatives of *H. crispa* Kunitz-type superfamily can substantially expand and, possibly, deepen our current understanding of the evolutionary relationships of these unique polypeptides, investigation into the role different protease inhibitors and their biological targets play is necessary for finding new candidates with therapeutic potential.

In this paper, using a combination of traditional isolation and structural protein chemistry approaches, we investigate kinetic and thermodynamic features and a functional activity, in particular, specificity to serine proteases, for two new native representatives of the recently discovered HCRG subfamily from *H. crispa*. Herein we also test for the first time an anti-inflammatory activity of sea anemone Kunitz-type polypeptides through their influence on some inflammation mediators *in vitro*.

## 2. Results and Discussion

### 2.1. Isolation and Polypeptide Amino Acid Sequence Determination

**Figure 1:**
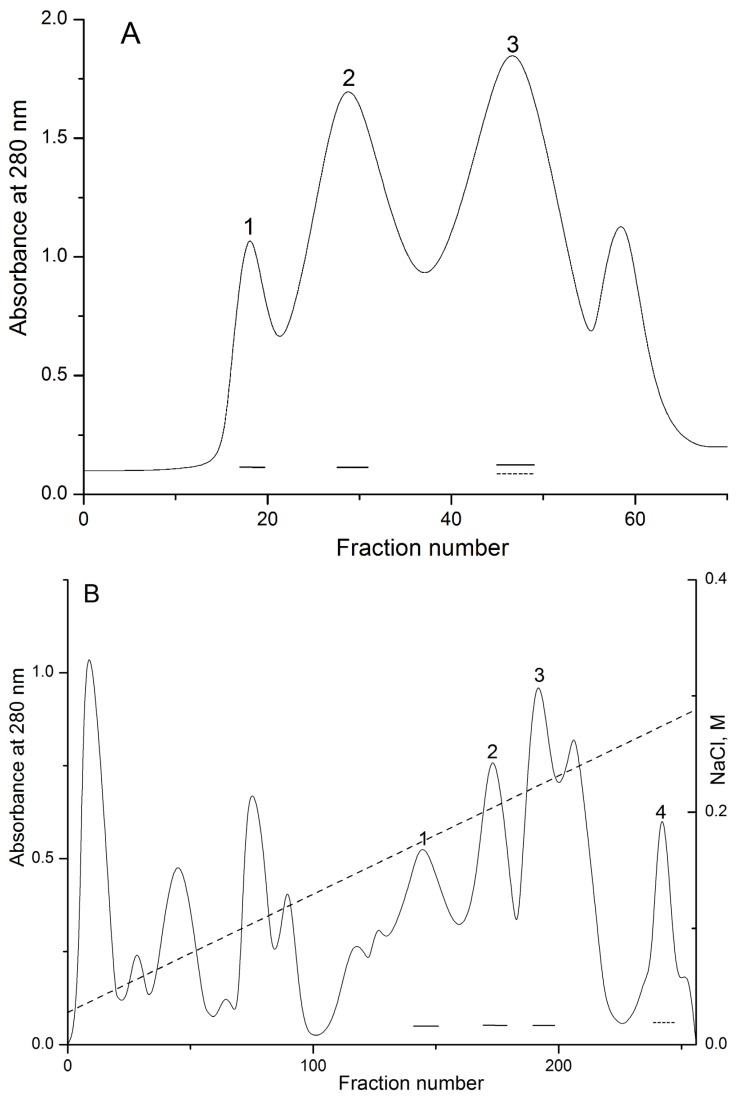

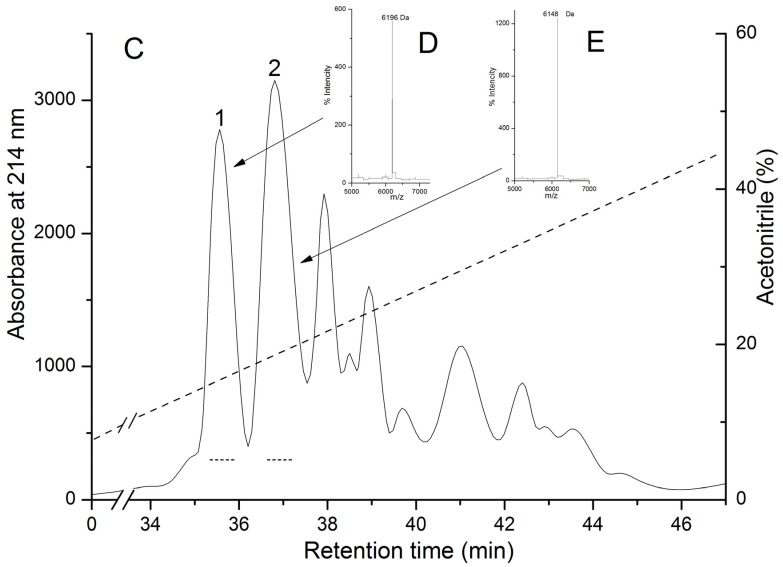
Elution profiles of *H. crispa* polypeptides at various stages of chromatographic purification. (**A**) Gel filtration chromatography of polypeptides contained in 80% acetone powder on column with Akrilex P-4; (**B**) Subsequent cation-exchange chromatography of active fraction polypeptides (Figure 1A, peak 3) on column with cellulose CM-32; (**C**) RP-HPLC performed on Nucleosil C_18_ column of polypeptides (Figure 1B, peak 4) desalted on an Akrilex P-4 column. Fraction with hemolytic and trypsin inhibitory activities are accentuated by solid and dotted lines, respectively. MALDI-TOF/MS spectrums and molecular masses of HCRG1 (**D**) and HCRG2 (**E**) after RP-HPLC are shown in the inset. Chromatography conditions are described in the Experimental Section (Methods).

Biologically active polypeptides from the water extract of the sea anemone *H.* *crispa* possess hemolytic activity (pore-forming toxins or actinoporins) and trypsin inhibitory activity (Kunitz-type protease inhibitors) were precipitated with 80% acetone as was described in [34]. Gel filtration of polypeptides contained in the water solution of acetone powder on Akrilex P-4 column gave three protein fractions (Figure 1A, peaks 1–3) with biological active polypeptides. The fractions corresponding to peaks 1 and 2 were found to exhibit high hemolytic activity; peak 3 exhibited both light hemolytic and trypsin inhibitory activity. Polypeptides from peak 3 were pooled, lyophilized, and purified by cation-exchange chromatography on a CM-32 cellulose column (Figure 1B), with peaks 1–3 resulting in hemolytic activity and peak 4 only in trypsin inhibitory activity. According to MALDI-TOF/MS analysis, these fractions contained polypeptides with molecular masses of approximately 6 kDa. As a result of RP-HPLC, the two individual polypeptides (Figure 1C, peaks 1 and 2, respectively) possessing trypsin inhibitory activity were isolated. According to MALDI-TOF/MS data, the polypeptides’ molecular masses were 6196 and 6148 Da, respectively (Figure 1C–E).

Due to the fact that all Kunitz-type inhibitors from *H. crispa* have three disulfide bonds [23,24,25,26,29,30], polypeptides HCRG1 and HCRG2 were alkylated. The molecular masses of modified polypeptides were higher than those of native polypeptides by 630 Da (6824 Da for HCRG1 and 6776 Da for HCRG2). This indicates that each molecule contains six cysteine residues, which apparently form three disulfide bonds.

**Figure 2:**
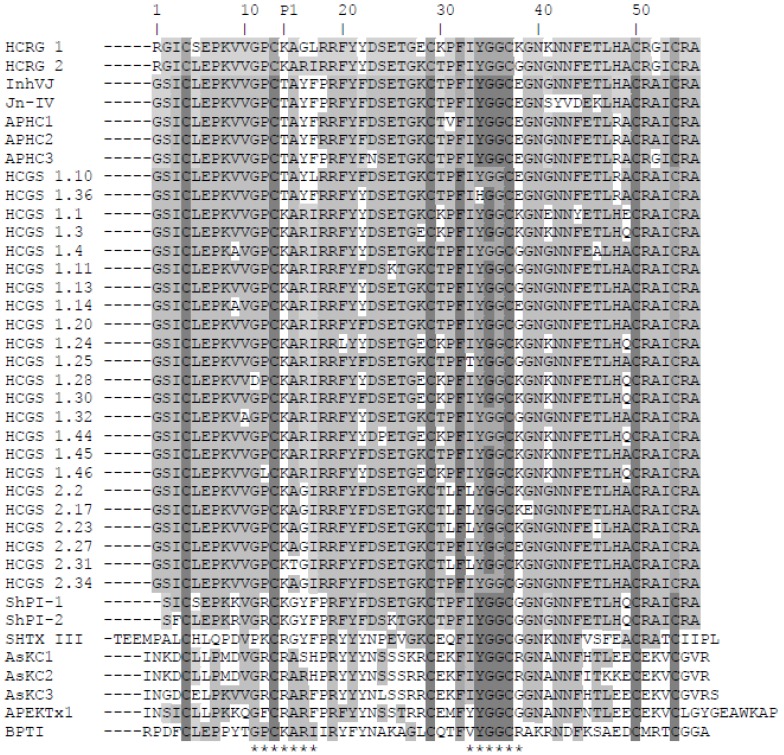
Alignment of sea anemone Kunitz-type polypeptides’ amino acid sequences. Native HCRG1, HCRG2, InhVJ [26], and Jn-IV [23], APHC1, APHC2, and APHC3 [29,30], and representatives of HCGS-polypeptides with P1Thr or Lys from the combinatorial library, obtained from *H. crispa* cDNA [15]; ShPI-1 and ShPI-2 from *Stichodactyla helianthus* [27,28]; SHTX III from *Stichodactyla haddoni* [17]; AsKC1, AsKC2, and AsKC3 from *Anemonia* *sulcata* [16]; APEKTx1 from *Anthopleura elegantissima* [18]; BPTI from *Bos taurus* [35]. P1—amino acid residue of inhibitor reactive center. The asterisks below the sequence of BPTI indicate the contact sites with serine proteases. Identical and conservative residues are indicated by dark and light gray colors.

After digestion of modified polypeptides by *Staphylococcus aureus* V8 endoprotease Glu-C and comparison of peptide sequences with the known primary structures of the inhibitors from *H. crispa* [15,23,26,29,30], the complete amino acid sequences of HCRG1 and HCRG2 (56 amino acids) were deduced (Figure 2).

Both HCRG1 and HCRG2 polypeptides have the Lys14 residue at the P1 position, which is part of the canonical binding loop (positions 11–17) essential for formation of a stable complex with serine proteases’ active center through electrostatic and hydrophobic interactions [36]. This is also found in BPTI and the majority of sea anemone inhibitors from *H. crispa* [15]. Having Thr at the P1 position rather than Arg or Lys has led to the simultaneous neofunctionalization of these polypeptides, allowing them to modulate the TRPV1 receptor [29,30,37] or inhibit exclusively trypsin-like proteases [23,25,26]. An interesting feature of both polypeptides is the presence of a positively charged Arg residue on the *N*-terminus of the molecule, which are unique to HCRG1 and HCRG2 polypeptides. Comparative analysis of the amino acid sequences of the two new polypeptides along with all known sequences of the inhibitors isolated from *H. crispa* (Figure 2) revealed a high degree of sequence similarity (from 69% to 95% of identity). This indicates that HCRG1 and HCRG2 are the isoforms of Kunitz-type polypeptides produced by sea anemone *H. crispa*, and may be classified as the first representatives of the new polypeptide subfamily that is encoded by the RG gene. According to the previous results of analyses of 5′-RACE cDNA of *H. crispa*, three mature polypeptides named S11, S12, and S13 with an *N*-terminal Arg1 were obtained [15]. Only the *N*-terminal sequence of HCRG2 (1–30 amino acid) was found to be absolutely identical with the mature polypeptide S11. Therefore we suppose that the *H. crispa* sea anemone produces a great variety of not only HCGS polypeptides but of HCRG polypeptides, which both form a combinatorial library.

It is important to note that HCRG2 has an identical amino acid sequence fragment (-GPCKARI-) with BPTI at the main contact site of the molecule, while HCRG1 has two substitutions at this region, Arg16 to Gly and Ile17 to Leu. Beside this, there are some basic substitutions in the 1, 5, 16, 17, 28, 30, 38, and 41 positions of the amino acid sequences of native HCRG1, HCRG2, and HCGS polypeptides, each of which can obviously influence the interaction of the polypeptides with trypsin [14,38,39]. Furthermore, it should be mentioned that practically 100% of the identity of the tetrapeptide (34–37 amino acid) is in the polypeptides’ weak contact sites (33–38 amino acid), with the exception of HCGS 1.36 with His34 residue (Figure 2).

### 2.2. Interaction of HCRG1 and HCRG2 with Serine Proteases

The basis of the Kunitz-type polypeptides’ inhibitory activity is a specific interaction between the P1 amino acid residue of the canonical binding loop with the S1 pocket of trypsin-like proteases’ active center [36]. In addition, there is scientific evidence that contribution to the association energy of the inhibitor-trypsin complex is likely made by amino acid residues of both sites at positions 11–17 of the inhibitor reactive site or the main contact site as well as at positions 33–38 of the weak contact site [26,38,39,40].

The values of the inhibition constants (*K*_i_) of trypsin calculated by the Dixon method were 2.8 × 10^−8^ M for HCRG1 and 5.0 × 10^−8^ M for HCRG2. These constants fall within the predictable range based on other *H. crispa* Kunitz-type polypeptides, analgesic APHC1–APHC3, and specific inhibitor InhVJ (1.0 × 10^−6^, 0.9 × 10^−6^, 5.0 × 10^−7^ and 7.38 × 10^−8^ M, respectively) [26,29,30]. The constants of inhibition of all native Kunitz-type inhibitors from the different sea anemone species with positively charged Arg or Lys at the P1 position lie in the range of values 10^−7^–10^−10^ M [16,17,18,19,20,21,27,28], whereas the polypeptides with P1Thr range from 10^−6^ to 10^−9^ M (Table 1) [25,26,29,30]. Nevertheless, on the similar Lys14 at the P1 position of both polypeptides, the *K*_i_ values of trypsin for the HCRG1 and HCRG2 were higher than ShPI-1 (*K*_i_ = 1.1 × 10^−10^ M) from *S. helianthus* [27]. ShPI-1 has broad protease specificity and inhibits not only serine, but also cysteine and aspartate proteases. The main structural difference between HCRG-polypeptides and ShPI-1 is the substitution of Pro12 (the numbering is based on the sequence of HCRG-polypeptides) to Arg12. It was deduced that besides Lys14, the prominent role in stabilization of the complex rShPI-1A-trypsin is attributed to Arg12 [38]. The same substitution is observed in bifunctional Kunitz-type polypeptides, kalicludines (AsKC1, AsKC2 and AsKC3), strong inhibitors of trypsin (*K*_i_ = 3 × 10^−10^ M), and blockers of voltage-gated potassium channels (Table 1) [16]. Besides the substitutions at positions 12 and 14, different ones are observed at positions 16 and 17 of the inhibitors’ main contact site (Table 1). They can also influence the interaction with trypsin [12,36]. It should be noted that HCRG2 has Arg16, similar to BPTI, AsKC2, AsKC3, and APEKTx1. There is a synonymous substitution of Ile in HCRG2 (like the one in BPTI) to Leu in HCRG1 at position 17. The conservation of the amino acids at position 34–37 of the weak contact site is nearly 100%; however, there is one substitution at position 38 that influences the molecular affinity to serine proteases [39]. It should be emphasized that there is no direct correlation between the inhibition constants’ values and the contact sites’ amino acids. For example, the *K*_i_ values of InhVJ with Thr14 and Tyr16 to trypsin are retained about 10^−8^ M, and APHC1–APHC3 with the same amino acids in both sites have different *K*_i_ values. We hypothesized that the Kunitz-type trypsin inhibitory activity data, together with the analysis of polypeptides’ amino acid variability at the interface region, provide evidence that some residues out of the contact sites (such as Arg1 for HCRG1 and HCRG2, Glu45 for InhVJ [26], Ile19 for BPTI [18,39], and some others) influence protease–inhibitor complex stability as well as the appearance of a new function [18,29,30].

**Figure 3:**
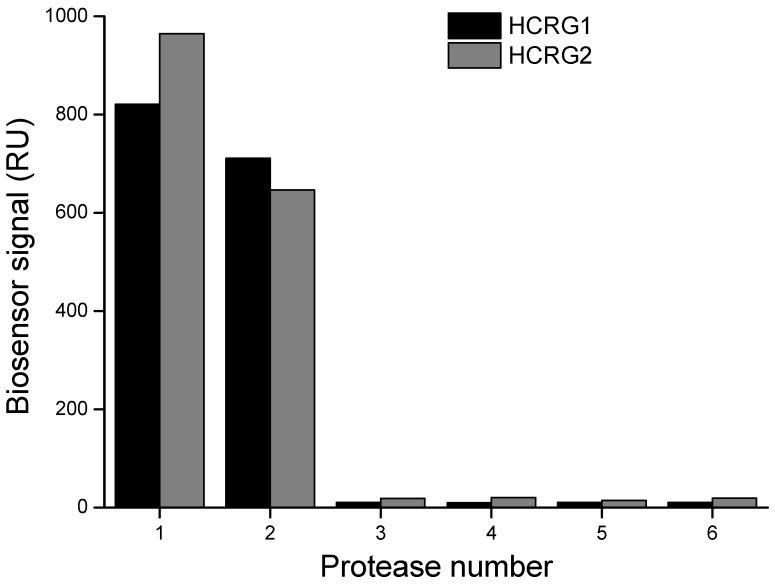
Interaction of the immobilized HCRG1 and HCRG2 with serine proteases: 1—α-chymotrypsin (20 nM), 2—trypsin (20 nM); 3—plasmin (23 nM); 4—kallikrein (20 nM); 5—thrombin (20 nM); 6—HBS-EP buffer. Measurements were carried out by the surface plasmon resonance (SPR) method at 25 °C.

The interaction of native HCRG-polypeptides with serine proteases (trypsin, α-chymotrypsin, thrombin, kallikrein, and plasmin) was also studied by the surface plasmon resonance (SPR) method. As seen in Figure 3, only trypsin and α-chymotrypsin at a very low concentration (20 nM) bound specifically with both inhibitors immobilized on a sensor chip.

**Table 1:** Comparison of amino acids at the contact sites of sea anemones’ protease inhibitors and their inhibitory activity against trypsin.

Sea Anemone	Peptide	Main Contact Site							Weak Contact Site						Inhibitory Activity against Trypsin
	Number of aa	11	12	13	14	15	16	17	33	34	35	36	37	38	
*H. crispa*	HCRG1	G	P	C	**K**	A	**G**	**L**	I	Y	G	G	C	**K**	28 *
	HCRG2	G	P	C	**K**	A	**R**	**I**	I	Y	G	G	C	**G**	50 *
	InhVJ [26]	G	P	C	**T**	A	**Y**	**F**	I	Y	G	G	C	**E**	73.8 *
	Jn-IV [23]	G	P	C	**T**	A	**Y**	**F**	I	Y	G	G	C	**E**	9.6 *
	APHC1 [29]	G	P	C	**T**	A	**Y**	**F**	I	Y	G	G	C	**E**	1000 *
	APHC2 [30]	G	P	C	**T**	A	**Y**	**F**	I	Y	G	G	C	**E**	900 *
	APHC3 [30]	G	P	C	**T**	A	**Y**	**F**	I	Y	G	G	C	**E**	500 *
*S. helianthus*	SHPI-1 [27]	G	**R**	C	**K**	**G**	**Y**	**F**	I	Y	G	G	C	**G**	0.11 *
*A. sulcata*	AsKC1 [16]	G	**R**	C	**R**	A	**S**	**H**	I	Y	G	G	C	**R**	<30 *
	AsKC2 [16]	G	**R**	C	**R**	A	**R**	**H**	I	Y	G	G	C	**R**	<30 *
	AsKC3 [16]	G	**R**	C	**R**	A	**R**	**F**	I	Y	G	G	C	**G**	<30 *
*S. haddoni*	SHTX III [17]	**P**	**K**	C	**R**	**G**	**Y**	**F**	I	Y	G	G	C	**G**	203 IU/mg **
*A*. *elegantissima*	APEKTx1 [18]	G	**F**	C	**R**	A	**R**	**F**	Y	Y	G	G	C	**G**	120 *
*B. taurus*	BPTI [35]	G	P	C	**K**	A	**R**	**I**	V	Y	G	G	C	**R**	0.00006 *

* Measurement of activity in terms of *K*_i_ value (nM); ** Measurement of activity in terms of inhibitory units (IU)/mg, where 1 IU is the amount of protein that inhibits one unit of enzyme.

**Figure 4:**
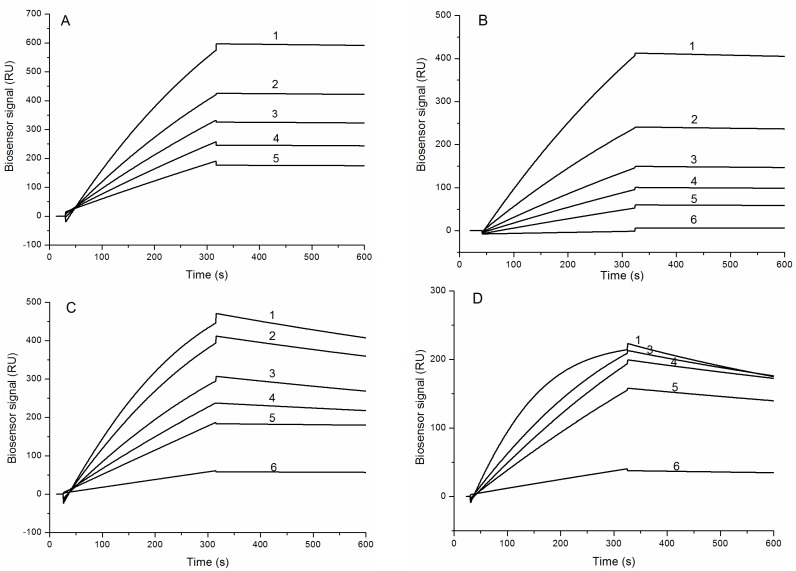
Binding sensograms of the immobilized protease inhibitors with trypsin and α-chymotrypsin at 25 °C. Interaction of HCRG1 (**A**) and HCRG2 (**B**) with trypsin: 1-21.7 nM, 2-16.2 nM, 3-10.85 nM, 4-7.6 nM, 5-5.4 nM, 6-1.1 nM. Interaction of HCRG1 (**C**) and HCRG2 (**D**) with α-chymotrypsin: 1-20 nM, 2-15 nM, 3-10 nM, 4-7 nM, 5-5 nM, 6-1 nM.

The binding sensograms of HCRG1 and HCRG2 along different concentrations of trypsin and α-chymotrypsin indicate that trypsin bound strongly to both inhibitors with almost no dissociation during the wash phase (Figure 4A,B), while the bonding of α-chymotrypsin is slow and with meaningful dissociation (Figure 4C,D).

Dissociation constants (*K*_D_) of the complexes HCRG1/TRP and HCRG2/TRP were 2.2 × 10^−10^ M and 6.8 × 10^−10^ M, respectively (Table 2). At the same time, the binding of α-chymotrypsin with HCRG1 and HCRG2 was about an order of magnitude lower than that with trypsin (*K*_D_ = 1.8 × 10^−9^ M and 1.6 × 10^−9^ M, respectively). The difference in *K*_D_ values for the inhibitor/trypsin and inhibitor/α-chymotrypsin complexes apparently originated from the values of the dissociation rate constants of these complexes (Table 2).

Previously we studied the interaction of the atypical inhibitor InhVJ (P1Thr) with proteases by the SPR method and demonstrated that InhVJ is a specific inhibitor of trypsin and α-chymotrypsin, and it had no inhibitory effect on plasmin, thrombin, kallikrein, cysteine protease papain, and aspartic protease pepsin. The affinity of InhVJ to trypsin and α-chymotrypsin was weaker than that of the HCRG-polypeptides (*K*_D_ = 7.4 × 10^−8^ and 9.9 × 10^−7^ M, respectively) [41].

**Table 2:** The parameters of complex formation between protease inhibitors (HCRG1, HCRG2) and serine proteases (trypsin (TRP), α-chymotrypsin (ChTRP)).

Complex	*k*_a_, (M^−1^s^−1^)	*k*_d_, (s^−1^)	*K*_D_, (M)	ΔH, kJ/mol	−TΔS, kJ/mol	ΔG, kJ/mol
HCRG1/TRP	(1.7 ± 0.03) × 10^5^	(3.7 ± 0.2) × 10^−5^	(2.2 ± 0.1) × 10^−10^	33 ± 4	−88	−55
HCRG2/TRP	(1.1 ± 0.02) × 10^5^	(7.7 ± 0.3) × 10^−5^	(6.8 ± 0.3) × 10^−10^	39 ± 5	−92	−53
HCRG1/ChTRP	(3.6 ± 0.2) × 10^5^	(6.2 ± 0.1) × 10^−4^	(1.8 ± 0.1) × 10^−9^	28 ± 3	−77	−49
HCRG2/ChTRP	(6.6 ± 0.1) × 10^5^	(1.0 ± 0.01) × 10^−3^	(1.6 ± 0.03) × 10^−9^	10 ± 2	−60	−50

Where: *k*_a_—association rate constants, *k*_d_—dissociation rate constants, *K*_D_—dissociation constants, ΔG—changes in Gibbs energy, −TΔS—entropic term, and ΔH—changes in enthalpy.

In addition to kinetic constants, we determined the thermodynamic characteristics (ΔG, −TΔS, and ΔH) of intermolecular interactions between the protease inhibitors and serine proteases (Table 1). Changes in temperature (10–40 °C) revealed that both association and dissociation constants’ rates changed as expected, with an optimal range similar to what was described for the complexes InhVJ/TRP and InhVJ/ChTRP, with the temperature dependence of free energy change (ΔG) between HCRG1 and HCRG2 satisfactorily approximated by the first order polynomial [42]. The magnitude of ΔG was higher for the most stable complexes of HCRG-polypeptides with TRP and ChTRP and lower for the least stable complexes of InhVJ with these proteases.

The negative values of the entropic term (−TΔS) determined during the biosensor analysis favor the complex formation, while positive values of enthalpy term changes (ΔH) counteract this process. These effects may be attributed to the displacement of water molecules from hydrophilic sites of the protease–inhibitor interaction interface and conformational transitions in protease and/or desolvation of polar groups that occur upon inhibitor binding [42].

### 2.3. Structure Modeling

**Figure 5:**
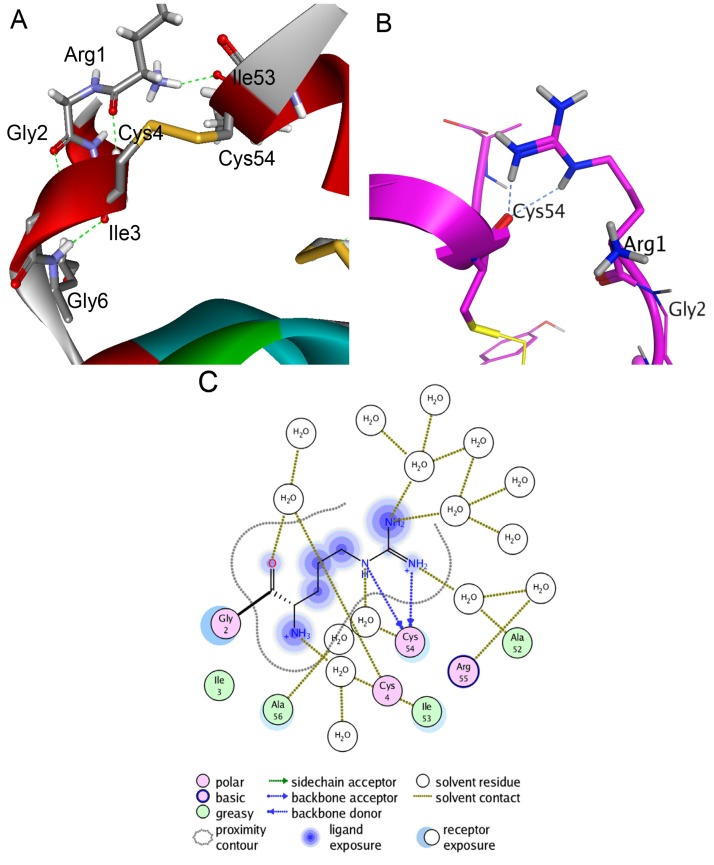
Molecular modeling of intramolecular interactions of HCRG2 *N*-terminal amino acid residue Arg1. Diagram was prepared with Discovery Studio Visualizer 3.0 (Accelrys Software Inc., San Diego, CA, USA). (**A**) The scheme of Arg1 intramolecular interactions after 60 ns MD simulations of HCRG2 in an aqueous environment at 300 K. The HCRG2 spatial structure fragment is represented as ribbon diagram and colored according to secondary structure elements. The side chains of amino acid residues participating in formation of hydrogen bonds between Arg1, Ile53, and Cys4 are shown as sticks; (**B**) Intramolecular interactions between the *N*- and *C*-terminal HCRG2 regions formed by the Arg1 side chain. Hydrogen bonds of Arg1 with Cys54 after 60 ns MD simulations of HCRG2 in an aqueous environment at 390 K. The HCRG2 spatial structure region is represented as a ribbon diagram, the Arg1 residue is shown as ball and sticks, and other amino acid residues participating in the formation of hydrogen bonds as sticks; (**C**) Schematic representation of direct hydrogen bonds and water-mediated contacts formed by Arg1 residue. Diagrams B and C were prepared using the MOE program (CCG).

To fulfill the structure-functional analysis of both native polypeptides and to analyze and interpret their properties and behavior observed during biochemical, kinetic, and thermodynamic studies, spatial structure models of HCRG1 and HCRG2 were constructed by homology modeling with the conformational features identified by Molecular Dynamics (MD) simulations. The modeling results indicated that the molecule skeleton of HCRG1 and HCRG2 was stabilized not only through S–S bonds, which were specific for the Kunitz fold, but also for the additional intramolecular H-bond formed by the Arg1 side chain and Ile53 (Figure 5A). Based on MD simulations (300 K in an aqueous solution), the energy contribution of this bond increased molecule stability from −4.3 to −11.4 kcal/mol due to the distance between the Cα atoms in Ile53 and the *N*-terminal amino acid residue Arg1 is reduced from 5.50 to 3.79 Å, and molecules of both polypeptides become more tightly packed. Additionally, high-temperature simulations (390 K) revealed that the H-bonds from this side chain may be rearranged without becoming dissociated, indicating the rigidity of the arrangement (Figure 5B) mediated by two hydrogen bonds with Cys54 (total contribution to the energy of the molecule is −3.4 kcal/mol) as well as by water-mediated contacts with Ala52, Ala56, Cys4, and Ile53 (Figure 5C). This interaction was absent for the HCGS polypeptides [15].

**Figure 6:**
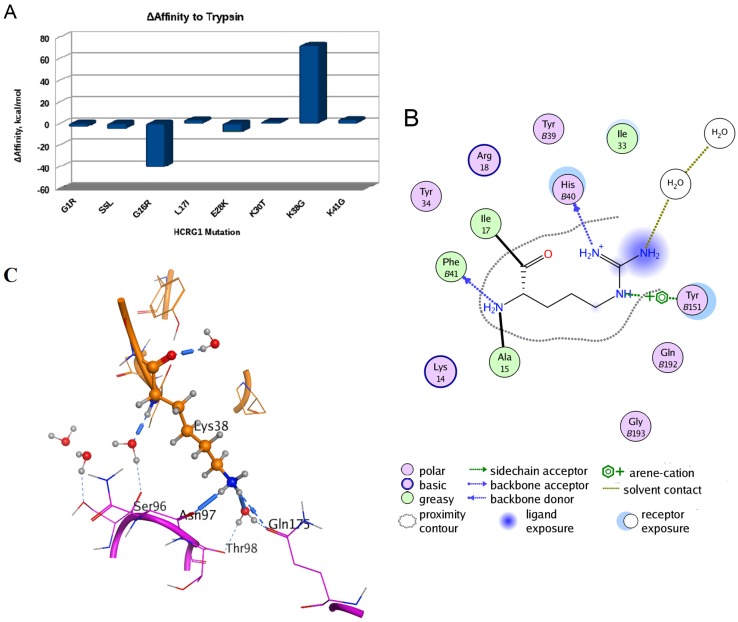
Computational mutagenesis of the HCRG1 and HCRG2 polypeptides’ affinity to trypsin. (**A**) Diagram of the binding affinity change of the polypeptide HCRG1 to trypsin upon amino acid mutations at positions 1, 5, 16, 17, 28, 30, 38, and 41 (obtained by MOE Protein Design tool [43]); (**B**) Schematic presentation of hydrogen bonds and arene-cation bonds formed by Arg16 residue. The numbers of trypsin residues are marked with the letter “B”; (**C**) The inter- or intra-molecular hydrogen bonds formed by the Lys38 residue (represented as ball and sticks). The spatial structure fragments are shown as a ribbon diagram, and the neighboring amino acid residues participating in the formation of hydrogen bonds as sticks.

To understand a possible effect of substitutions of HCRG1 and HCRG2 amino acid residues localized at the interface area on their affinity to trypsin, the structure models of the polypeptide complexes with the protease were generated by the molecular docking method. *In silico* mutagenesis of residues discriminating HCRG1 and HCRG2 polypeptides (at positions 5, 16, 17, 28, 30, 38, and 41) showed that substitutions made a multidirectional contribution to the polypeptides’ affinity to trypsin, with the most significant contribution at 16 and 38 (Figure 6A).

Ultimately, this highlighted that any substitution of positively charged and volumetric Arg16 residue at position 16 for HCRG2 makes a positive contribution to the trypsin binding affinity with the minimal one for Arg16 substitution to Lys (ΔAffinity is +2.144 kcal/mol). This result agrees well with the data, showing that the enrichment of the main contact site of Kunitz-type polypeptides with positively charged residues raises the effectiveness of protease inhibition [18,44]. Replacement of Gly16 to Arg, which is typical for HCRG2, apparently promotes the formation of a tighter complex with the enzyme; the contribution to ΔAffinity is −40.514 kcal/mol. The Gly16 backbone of HCRG1 forms a H-bond with Phe41 of trypsin, as was observed for Tyr15 and Met17 (both equal to Gly16 of HCRG1) of rShPI-1A and inhibitor domain of Alzheimer’s amyloid beta-protein precursor (APPI), respectively (PDB IDs 3M7Q and 1TAW, respectively). By contrast, Arg16 of HCRG2 stabilizes this complex, not only by backbone H-bonding, but, similarly to Arg17 of BPTI, also through additional side chain hydrogen bonding with His40 [38] as well as by arene-cation interaction with Tyr151 of trypsin (Figure 6B). On the other hand, substituting Lys38 to Gly at the weak contact site of the HCRG2 polypeptide contributes to the weakening of the inhibitor forces of interaction with trypsin (+72.3435 kcal/mol, Figure 6A). The small neutral side chain of Gly38 located on the border of the HCRG2–trypsin interface is not capable of supporting the structure of this complex by tight hydrophobic contacts and by direct or water-mediated hydrogen bonds either with trypsin Asn97 backbone or with Gln175 side chain, while such bonds are formed by long and positively charged Lys38 side chains of HCRG1 (Figure 6C) and Arg39 (equal to Lys38 of HCRG1) of BPTI (PDB ID 2FTL).

The best-studied and most potent inhibitor of serine proteases of the Kunitz family is BPTI (*K*_i_ in the range 10^−11^–10^−14^ M). It has been well established that Lys14 (the numbering is based on the sequence of HCRG-polypeptides), Arg38, and Ile18 greatly contribute to the extremely high affinity of BPTI to serine proteases [18,39,45]. Notably, all known Kunitz polypeptides from sea anemones (Figure 2) have Pro18 or Arg or Thr18 instead of Ile18 in BPTI. This contributes to additional interaction with the side chain of Tyr39 of trypsin, resulting in a weaker affinity to trypsin. Consequently, the substitutions in HCRG1 and HCRG2 at positions 16, 18, and 38 may explain the lower serine protease inhibitory activity and differences of the kinetic parameters of the interaction (*K*_i_ and *K*_d_) with trypsin, which we have observed experimentally.

### 2.4. Determination of HCRG1 and HCRG2 Anti-Inflammatory Activity

A number of reports are devoted to the prospect of using Kunitz-type protease inhibitors (aprotinin [32], bikunin [46], hepatocyte growth factor activator inhibitor (HAI) [47], and tissue factor pathway inhibitor (TFPI) [48]) with different anti-inflammatory action in the treatment of inflammatory pathologies [31]. Moreover, a Kunitz-type serine protease inhibitor, SBTI from soybeans [49,50], has a protective effect on lipopolysaccharide (LPS)-induced inflammation through inhibiting the enhanced production of proinflammatory molecules including such mediators as interleukin-1β (IL-1β), interleukin-6 (IL-6) [50], and tumor necrosis factor-α (TNF-α) [51,52]. This prompts the search for new sources of inhibitors and study of their therapeutic potential, in particular the anti-inflammatory action.

The study of the anti-inflammatory effects of Kunitz-type polypeptides from the sea anemone *H. crispa* interacting with proteases involved in many inflammatory processes (trypsin, elastase, cathepsin G, and others) [27], as well as transduction of pain signals through the vanilloid TRPV1 receptor [29,30,37], is of great interest both fundamentally and from an applied point of view. Analgesic polypeptides APHC1–APHC3 possess anti-inflammatory activity [53] and the significant analgesic effect produced without hyperthermia [54]; this is a very useful pharmacological feature of analgesic compounds. Previously we have found that the two native Kunitz-type inhibitors, RmIn I (GICSEPIVVGPCKAG-) and RmIn II (GSTCLEPKVVGPCKA-), which have a high degree of identity with HCRG-polypeptides, possess antihistamine activity *in vivo* [24]. This was demonstrated by an increase in desensitization over time in response to the injection of inhibitors prior to the administration of histamine [24]. This effect could be due to the RmIn I and RmIn II inhibitory effect on mediators that cause inflammation in the cells, as well as on the H1-histamine receptor or inflammatory proteases.

Recently we have shown that recombinant polypeptide HCGS 1.20 from the HCGS subfamily with P1Lys possesses an antihistamine activity through blocking of H1-histamine receptors as well as an anti-inflammatory activity through inhibiting of NO-synthase expression [33]. In the polypeptide HCRG1 and HCRG2, and HCGS 1.20 sequences some substitution are observed: Tyr22 to Phe, Gly52 to Ala for both of them, and Leu5 to Ser, Gly16 to Arg, Leu17 to Ile, Glu28 to Lys, and Lys30 to Gly for HCRG 1 (Figure 2). BPTI possesses an anti-inflammatory activity but provokes allergic effects that limit its operation [32]. Due to this fact, it is important to search for new representatives of Kuntz-type polypeptides that possess an anti-inflammatory activity without such side effects.

**Figure 7:**
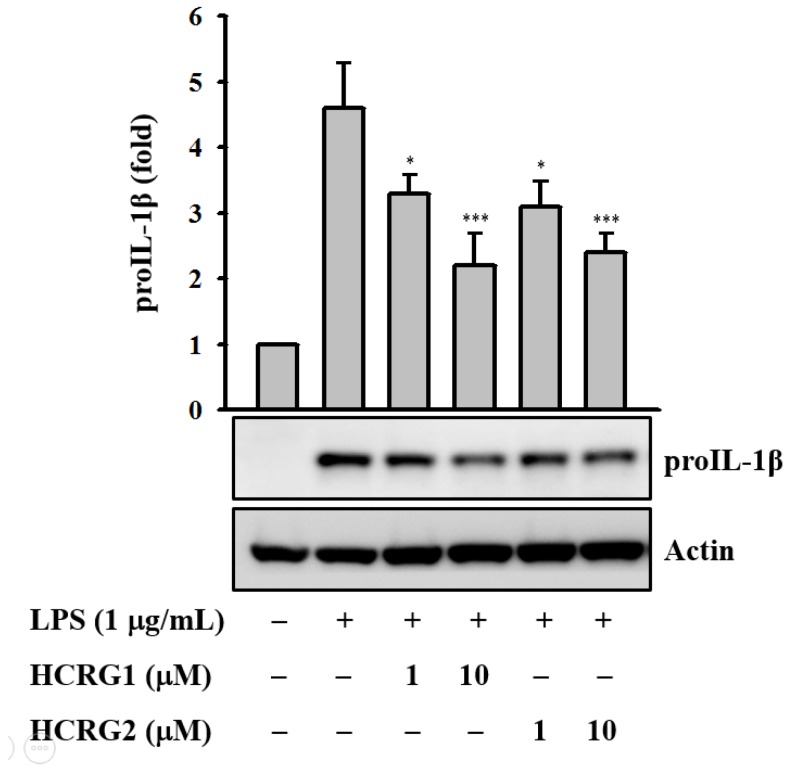
Kunitz-type trypsin inhibitors (HCRG1 and HCRG2) prevent proIL-1β protein expression. J774A.1 macrophages were pretreated with HCRG1 and HCRG2 (1 and 10 µM) or medium for 30 min and subsequently challenged with lipopolysaccharide (LPS) (1 µg/mL) for 6 h. The Western blot results are representative of those obtained in three different experiments, and the histograms are presented as the change in the ratio relative to proIL-1β compared to the control group. * and *** indicate a significant difference at the level of *p* < 0.05 and *p* < 0.001, respectively.

In this study we found that HCRG1 and HCRG2 dose dependently reduced IL-1β precursor (proIL-1β) expression levels in LPS-activated J774A.1 macrophages (Figure 7). In the same condition, the polypeptides did not show cytotoxicity to the cells.

**Figure 8:**
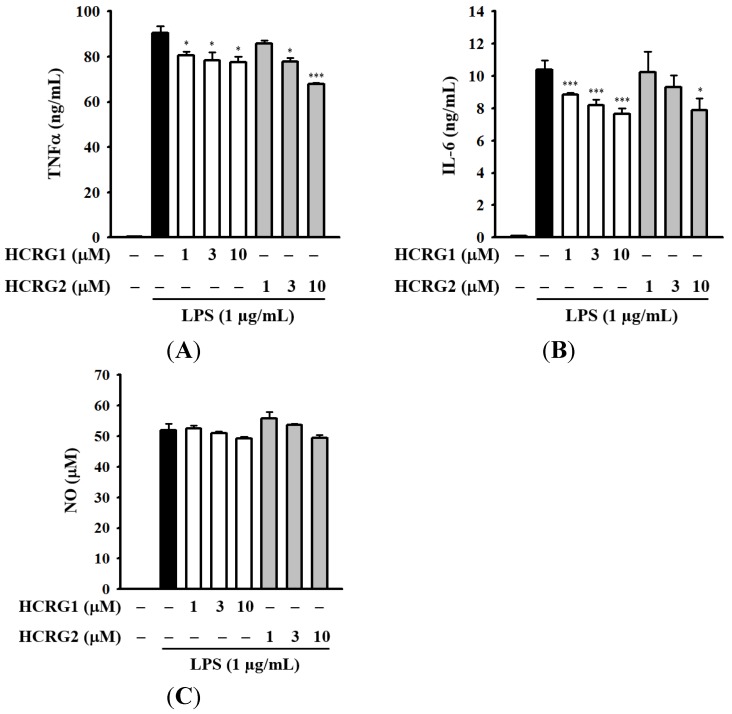
Inhibition of LPS-induced expression of inflammatory mediators: tumor necrosis factor-α (TNF-α) (**A**); interleukin-6 (IL-6) (**B**); and NO (**C**) by Kunitz-type polypeptides HCRG1 and HCRG2. RAW 264.7 macrophages were treated with and without HCRG1 and HCRG2 at the indicated concentrations for 30 min, followed by LPS (1 μg/mL) stimulation for 24 h. The levels of TNF-α and IL-6 in the culture medium were measured by enzyme-linked immunosorbent assay (ELISA). The levels of nitric oxide (NO) in the culture medium were measured by Griess reaction. The data are expressed as the means ± SD for three separate experiments. * and *** indicate a significant difference at the level of *p* < 0.05 and *p* < 0.001, respectively.

We further analyzed the effect of Kunitz-type polypeptides HCRG1 and HCRG2 on LPS-induced TNF-α secretion in RAW 264.7 macrophages. As shown in Figure 8A, both HCRG1 and HCRG2 decreased the TNF-α secretion in LPS-activated RAW 264.7 macrophages. It has been demonstrated that TNF-α mediates the production of some cytokines during inflammation, in particular the production of IL-1β and IL-6 [55]. As expected, LPS significantly induced large increases in IL-6 secretion in RAW 264.7 macrophages (Figure 8B). Both HCRG1 and HCRG2, under the same experimental conditions, markedly reduced LPS-induced IL-6 secretion (Figure 8B). However, it should be noted that HCRG1 and HCRG2 were not able to reduce LPS-induced nitric oxide (NO) generation in contrast to HCGS 1.20 (Figure 8C). To evaluate the mechanism of anti-inflammatory activity of HCRG-polypeptides and functionally significant amino acid residues interacting with proinflammatory molecules, new experimental data are required.

## 3. Experimental Section

### 3.1. Materials

LPS (from *Escherichia coli* 0111:B4) and actin antibody were purchased from Sigma (St. Louis, MO, USA). Mouse IL-6 and TNF-α ELISA kits were purchased from R&D Systems (Minneapolis, MN, USA). IL-1β antibody was purchased from Santa Cruz Biotechnology, Inc. (Santa Cruz, CA, USA).

### 3.2. Methods

#### 3.2.1. Isolation and Polypeptide Amino Acid Sequence Determination

Specimens of the sea anemone *H. crispa* (=*R. macrodactylus*) were collected in the coral reefs of the Seychelles during a marine expedition aboard the research vessel *Academik Oparin*. Dr. C.D. Grybelniy (Zoological Institute of the Russian Academy of Sciences, Saint Petersburg, Russia) confirmed the identity of the species.

The acetone precipitation of the polypeptides from the sea anemone water extract was previously described in detail [34]. Gel filtration chromatography of the polypeptides of *H. crispa* contained in 80% acetone powder was performed on a column (3 cm × 100 cm) using Akrilex P-4 (Reanal, Budapest, Hungary) equilibrated with 0.01 M ammonium-acetate buffer, pH 6.0. The polypeptides were eluted with the same buffer at a flow rate of 18 mL/h. The fractions of the third peak (Figure 1A) with trypsin inhibitory activity were concentrated and the polypeptides were chromatographed on a column (2.2 cm × 50 сm) using cellulose CM-32 (Whatman, Maidstone, UK) equilibrated with 0.01 M ammonium-acetate buffer, pH 6.0. The polypeptides were eluted in a linear gradient of NaCl concentration (0–0.5 M) in the same buffer at a flow rate of 30 mL/h (4 °C). Fractions with trypsin inhibitory activity (Figure 1B, peak 4) were collected and desalted on a column using Akrilex P-4 (3 cm × 30 cm) equilibrated with 0.01 M ammonium-acetate buffer, pH 6.0. The polypeptides were eluted with the same buffer at a flow rate of 20 mL/h (4 °C). The obtained active fractions were then purified by RP-HPLC on a Nucleosil C_18_ column (4.6 cm × 250 mm; Sigma Aldrich, St Louis, MO, USA) in a linear gradient of acetonitrile concentration (10%–70%) in 0.1% trifluoroacetic acid (TFA) at a flow rate of 0.5 mL/min for 60 min (Figure 1C).

HCRG1 and HCRG2 were reduced and alkylated with 4-vinylpyridine as described in [24,26]. Alkylated polypeptides were digested with endoprotease Glu-C (Staphylococcus aureus V8-protease, Sigma, St Louis, MO, USA) 1:20 (w/w) in 100 µL of 100 mM ammonium bicarbonate (рH 8.0) at 37 °C for 5 h. Obtained peptides (five for the HCRG1 and four for the HCRG2) were separated and desalted by RP-HPLC.

#### 3.2.2. Physicochemical Characterization of HCRG1 and HCRG2

MALDI-TOF/MS spectra of HCRG1 and HCRG2 were recorded using an Ultra Flex III MALDI-TOF/TOF mass spectrometer (Bruker, Bremen, Germany) with a nitrogen laser (SmartBeam, 355 nm), reflector and potential LIFT tandem modes of operation. Sinapinic acid was used as a matrix. An external calibration was employed using a polypeptide sample [26] with *m*/*z* 6107 and its doubly-charged variant at *m*/*z* 3053.

The amino acid sequences of HCRG1 and HCRG2 were determined on an automated sequencer protein <Procise 492 Clc> (Applied Biosystems, Foster City, CA, USA). Homology sequence analysis was carried out using protein databases and BLAST programs [56].

The protein sequence data reported in this paper will appear in the UniProt Knowledgebase under the accession numbers C0HJU6 for HCRG1 and C0HJU7 for HCRG2. The accession numbers of the other polypeptide sequences are InhVJ (UniProt KB P0DMJ5), Jn-IV (UniProt KB P16344), APHC1, APHC2, and APHC3 (UniProt KB B2G331, C0HJF4, C0HJF3); ShPI-1 (UniProt P31713); SHTX III (UniProt KB/Swiss-Prot B1B5I8); AsKC1, AsKC2, and AsKC3 (UniProt KB Q9TWG0, Q9TWF9, Q9TWF8); APEKTx1 (UniProt KB P86862); and BPTI (Uniprot 00974).

#### 3.2.3. Hemolytic Activity

The hemolytic activity of the polypeptide fractions was tested with human erythrocytes in a medium containing 0.9% NaCl, 1 mM KCl, 10 mM glucose, 5 mM Tris-HCl, pH 7.4 after centrifugation. Following the final centrifugation the erythrocytes were resuspended to 0.7% hematocrit. Polypeptides were mixed with erythrocyte suspension and incubated at 37 °C for 30 min, chilled briefly, and centrifuged. The level of hemoglobin in the supernatant was measured spectrophotometrically at 540 nm. The lysis of 0.7% erythrocyte suspension with 10 μL of 10% NaCl solution was taken as 100% hemolysis (corresponding to an absorbance of 0.5 at a wavelength of 540 nm).

#### 3.2.4. Trypsin Inhibitory Activity

The trypsin inhibitory activity of the polypeptides was tested through the standard procedure [57] using *N*α-benzoyl-d,l-arginine p-nitroanilide (BAPNA) as a substrate.

Determination of the trypsin inhibition constants of HCRG1 and HCRG2 was performed according to the method of Dixon [58] using substrate (BAPNA) concentrations of 133 and 65 mM. The enzyme concentration in the reaction mixture is 215 nM. Concentrations of the tested polypeptides ranged from 0 up to 3.2 mM. The constants were calculated based on the results of three parallel experiments. Computational error limits are in the range of 0.1%–0.5%.

#### 3.2.5. SPR Measurements

The study of the interaction of serine proteases with HCRG1 and HCRG2 was performed on surface plasmon resonance (SPR) biosensor Biacore T200 (GE Healthcare Bio-Sciences AB, Uppsala, Sweden) running under the program “Biacore T200 Control v.1” with data evaluation using “Biacore Evaluation v.1”.

All binding assays were performed using Biacore CM5 sensor chips with a carboxymethylated dextran matrix. The covalent immobilization of protease inhibitors HCRG1 and HCRG2 on the surface of the CM5 chip was performed using the standard EDC/NHS amino-coupling Biacore protocol [42]. Interaction of different proteases with the immobilized inhibitors was studied using the concentration 20 nM. In all of the experiments, the flow cell 1 (Fc1) without the immobilized polypeptides was considered as the reference cell for the correction of the signal responses. HBS (HEPES buffered saline-NaCl): 0.01 M HEPES, pH 7.4, 0.15 M NaCl (cat. no. BR-1003-69, GE Healthcare Bio-Sciences AB, Uppsala, Sweden) was used as the running buffer for the SPR assays. After each cycle of SPR measurement, the sensing surface was regenerated by the injection of 50 mM NaOH for 0.5 min at a flow rate of 50 μL/min. Changes in Gibbs free energy (ΔG) were calculated by the following equation:

ΔG = RTln*K*_D_,

where *K*_D_ is the dissociation constant. Changes in enthalpy (ΔH) and entropic term (−TΔS) were calculated from the linear equation:

ΔG = ΔH − TΔS,

using the liner approximation of temperature dependence of ΔG (Van’t Hoff diagram) [42].

#### 3.2.6. Cell Cultures

The murine macrophage cell lines RAW 264.7 and J774A.1 were obtained from American Type Culture Collection (Rockville, MD, USA) and cultured in an RPMI 1640 medium supplemented with 10% heat-inactivated fetal bovine serum and 2 mM l-glutamine (all from Life Technologies, Carlsbad, CA, USA) at 37 °C in the presence of 5% CO_2_.

#### 3.2.7. Detection of proIL-1β

J774A.1 macrophages were seeded in a 6-cm culture dish at a density of 1 × 10^6^ cells/dish/2 mL medium for 24 h. The cells were incubated for 30 min with and without polypeptides. The cells were then incubated for 6 h with and without 1 μg/mL LPS. The levels of proIL-1β in the culture medium were measured by Western blot. In brief, whole cell lysates were separated by SDS-PAGE and electro-transferred to a PVDF membrane. The membrane was incubated in blocking buffer (5% nonfat milk in PBS with 0.1% Tween 20) overnight at 4 °C. The membrane was incubated with IL-1β or actin antibody at room temperature for 2 h. After washing three times with wash buffer (PBS with 0.1% Tween 20), the membrane was incubated with an HRP-conjugated secondary antibody directly against IL-1β or the actin antibody. After washing, the membrane was developed by an enhanced chemiluminescence Western blot detection system. The results were quantified by densitometric analysis using ImageJ software. The densitometry fold change of each group was calculated by comparing the results with the control group. The band density is normalized to actin before fold change is calculated.

#### 3.2.8. Detection of TNF-α and IL-6

RAW 264.7 macrophages were seeded in 24-well plates at a density of 1 × 10^5^ cells/well/0.5 mL medium for 24 h. The cells were incubated for 30 min with and without polypeptides. The cells were then incubated for 24 h with and without 1 μg/mL LPS. The levels of TNF-α and IL-6 in the culture medium were measured by Enzyme-Linked Immunosorbent Assay (ELISA) according to the manufacturer’s protocol. In brief, 50 μL of biotinylated antibody reagent and 50 μL of supernatant were added to an anti-mouse TNF-α and IL-6 precoated stripwell plate and incubated at room temperature for 2 h. After washing the plate three times with washing buffer, 100 μL of diluted streptavidin-HRP (horseradish peroxidase) concentrate was added to each well and incubated at room temperature for 30 min. The washing process was repeated; then, 100 μL of a premixed tetramethylbenzidine substrate solution was added to each well and developed at room temperature in the dark for 30 min. Following the addition of 100 μL of stop solution to each well to stop the reaction, the absorbance of the plate was measured by a microplate reader at a 450 nm wavelength.

#### 3.2.9. Detection of NO

RAW 264.7 macrophages were seeded in 24-well plates at a density of 1 × 10^5^ cells/well/0.5 mL medium for 24 h. The cells were incubated for 30 min with and without polypeptides. The cells were then incubated for 24 h with and without 1 μg/mL LPS. The levels of NO in the culture medium were measured indirectly by analysis of nitrite levels using the Griess reaction.

#### 3.2.10. Statistical Analyses

All values are given as the mean ± SD. The data analysis was performed by one-way ANOVAs followed by a Scheffé test. * and *** indicate a significant difference at the level of *p* < 0.05 and *p* < 0.001, respectively, compared to the LPS alone group.

#### 3.2.11. Structure Modeling of HCRG Polypeptides and HCRGs–Serine Protease Complexes

The spatial structure models of HCRG1 and HCRG2 polypeptides were generated using Modeller 9.11 and Chimera 1.9 programs [59,60]. The atomic coordinates of ShPI-1 (PDB ID 1SHP) from the sea anemone *S. helianthus*, which is the only known spatial structure of a protease inhibitor to date, was established by 1H-NMR spectroscopy [61] and used as a template (the identity between ShPI-1 and HCRG1 or HCRG2 is 75% and 80%, respectively). The quality of the models was tested using a web server PROCHECK [62].

##### Protein–Protein Docking

Models of spatial structure of complexes HCRG1 and HCRG2 with the experimentally established structures of serine proteases have been constructed via “blind” molecular docking by the PIPER program with clusterization thought the instrumentality of ClusPro 2.0 server [63,64].

##### Molecular Dynamics Simulation

Computations of molecular dynamics simulations for HCRG-protease complexes were performed under conditions of constant pressure, 300 K or 300–390 K, and pH 7.0 for 60 ns in an Amber12EHT force field using the MOE program (CCG) [43]. Prior to molecular dynamic simulations, the whole system was equilibrated to reduce initial bad contacts. Equilibration consisted of energy minimization of the initial side chains’ position with fixed backbone atoms, followed by minimization with restrained carbon alpha atoms and a short molecular dynamics (100 ps). The results were obtained using the equipment of Shared Resource Center “Far Eastern Computing Resource” IACP FEB RAS.

##### Computational Mutagenesis

Computational mutagenesis of the HCRG1 and HCRG2 and their affinity to trypsin assessment were produced with the MOE Protein Design tool [43]. Computer simulation and theoretical studies were performed using cluster CCU “Far Eastern computing resource” FEB RAS.

## 4. Conclusions

Our results indicate that unique HCRG1 and HCRG2 are the first representatives of a new Kunitz-type polypeptide subfamily of a larger gene family that was identified previously [15]. The Arg residue presence at position 1, making these polypeptides different to other sea anemones’ inhibitor sequences, leads to an increase of polypeptide molecular stability. Due to the P1Lys at the center of the canonical binding loop of both polypeptides, they are more potent inhibitors of serine proteases, trypsin, and α-chymotrypsin than known representatives of the HCGS subfamily with P1Thr. High homology of HCRG1 and HCRG2 amino acid sequences with representatives of the HCGS subfamily, the presence of P1Lys residue, and some substitutions along the sequences indicate that these polypeptides can, in addition to their protease inhibitory activity, have a number of new biological targets. Besides trypsin inhibitory activity, HCRG1 and HCRG2 exhibit anti-inflammatory activity. Our study first demonstrated that Kunitz-type polypeptides from sea anemones dose dependently reduce IL-1β precursor (proIL-1β) expression levels in LPS-activated J774A.1 macrophages. Pretreatment of cells with these polypeptides reduces TNF-α and IL-6 secretion as well as proIL-1β expression in LPS-activated macrophages. We suppose that the discovery of anti-inflammatory properties of Kunitz-type polypeptides from *H. crispa* allows us not only to considerably expand the arsenal of exogenous research tools for the determination of inflammation molecular mechanisms but also to create new anti-inflammatory agents for direct or combined action. To establish the molecular mechanisms of interactions of polypeptides with their biological targets, additional experimental and simulation data is required; HCRG1 and HCRG2, may provide a template for future investigations into pharmacologically active protease inhibitors.
